# Validation of a modified Chinese‐language version of the Davos Assessment of Cognitive Biases Scale (MCL‐DACOBS) in a sample of Chinese patients with schizophrenia

**DOI:** 10.1002/brb3.3185

**Published:** 2023-08-10

**Authors:** Guangdong Chen, Ranli Li, Hongjun Tian, Xiaoyan Ma, Yun Sun, Feng Jia, Jing Ping, Ziyao Cai, Jingjing Zhu, Chuanjun Zhuo, Zhi Pan

**Affiliations:** ^1^ Department of Psychiatry, Children Center Wenzhou Seventh Peoples Hospital Wenzhou China; ^2^ Department of Psychiatry, MECT Center Tianjin Anding Hospital Tianjin China; ^3^ Key Laboratory of Sensor Information Processing Abnormalities in Schizophrenia (SIPAS‐Lab) Tianjin Fourth Center Hospital Nankai University Affiliated Tianjin Fourth Center Hospital Tianjin Medical University Affiliated Tianjin Fourth Center Hospital Tianjin China

**Keywords:** CFA, ICC, MCL‐DACOBS, reliability, validity

## Abstract

**Introduction:**

The Davos Assessment of Cognitive Biases Scale (DACOBS) is widely used to assess cognitive biases in patients who have schizophrenia. However, the lack of a modified Chinese‐language version of the DACOBS (MCL‐DACOBS) precludes Chinese schizophrenic patients from treatment aimed at normalizing cognitive biases, impacting their prognosis. Here, we aimed to produce a DACOBS for China and test the validity and reliability of the resultant MCL‐DACOBS.

**Methods:**

Eighteen researchers collaborated to develop the MCL‐DACOBS: A total of 15 researchers modified and translated the English version of the DACOBS, 1 native‐English‐speaking researcher back‐translated the scale, and 2 Chinese sinologists localized and optimized the language of the MCL‐DACOBS. Forty‐two volunteers checked the scale items’ comprehensibility, and the two sinologists performed further localization and optimization based on their feedback. The final version of the MCL‐DACOBS used in this study was thus derived from the harmonized English‐language version of the scale. Confirmatory factor analyses (CFAs) were used to examine the best latent structure of the MCL‐DACOBS. Cronbach's *α* and intraclass correlation coefficients (ICCs) were used to check the reliability. The discriminative ability of the MCL‐DACOBS was assessed according to the area under the receiver operating characteristic curve.

**Results:**

The CFA showed that all items loaded onto factors with loadings >0.400. A two‐factor structure showed a good model fit (root mean square error of approximation = .018, Tucker–Lewis index = .978, comparative fit index = .984). Promax rotation demonstrated that each item had a high factor load (0.432–0.774). Cronbach's *α* coefficient and ICC for the MCL‐DOCABS were .965 and .957, respectively, indicating that the scale has ideal reliability.

**Conclusion:**

The MCL‐DACOBS has good validity and good reliability, and its psychometric properties indicate that it is a valid tool for measuring cognitive biases in Chinese patients with schizophrenia.

## Graphical abstract

The psychometric properties of the Chinese‐language version of the Davos Assessment of Cognitive Biases Scale (DACOBS) were confirmed to be consistent with those previously described for other language versions of the DACOBS.The modified Chinese‐language version of the DACOBS was shown to be a valid instrument for measuring cognitive biases and limitations in Chinese patients with schizophrenia.

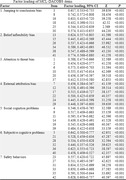

## 1 INTRODUCTION

In recent decades, cognitive impairment has been recognized as a core symptom of schizophrenia (Liddle, 2019; McCutcheon et al., 2020; Oliver et al., 2022; Robison et al., 2020) and received more attention than positive and negative symptoms in recent years (Bellani et al., 2019; Jones & Harvey, 2020; Kharawala et al., 2022; Mould et al., 2022; Sciortino et al., 2021). Cognitive bias is defined as a methodical orientation toward processing, selecting, appraising, and remembering specific information (Campbell et al., 2017; Landucci & Lamperti, 2021; O'Sullivan & Schofield, 2018; Ozdemir & Finkelstein, 2018). Cognitive biases can be classified into three types: attentional, interpretation, and memory (Mennen et al., 2019). Some cognitive biases have been shown to induce confirmatory bias or bias against disconformity evidence, jumping to conclusions, inflexibility of belief, and attributional biases (Bentall et al., 2001; Díaz‐Cutraro et al., 2021; Falcone et al., 2015; Garety et al., 2005; Moritz & Woodward, 2006). Cognitive biases affect several cognitive domains, including decision‐making/reasoning, attention, motivation, memory recall, and style of attribution of meaning (Beck et al., 2011). Increasing numbers of studies have been reporting cognitive biases that contribute to cognitive impairments in patients with schizophrenia, and cognitive bias and cognitive impairments appear to produce reciprocal deterioration and worsen disability (Frydecka et al., 2022; Hu et al., 2022; Navalón et al., 2021; Tang et al., 2022). Accordingly, assessing cognitive bias in patients with schizophrenia may enable the cognitive performance of patients with schizophrenia to be improved, thus improving their prognosis.

An ideal cognitive bias assessment tool can provide effective information for us to understand schizophrenia‐associated cognitive impairments. The Davos Assessment of Cognitive Biases Scale (DACOBS), which was developed by Professor van der Gaag et al. (2013), is used internationally to evaluate and measure specific cognitive biases in psychosis (Bastiaens et al., 2013; Livet et al., 2020, 2022; Pugliese et al., 2022; van der Gaag et al., 2013; Whitman et al., 2002). It consists of 42 items (Table 1), which are divided into seven 6‐item subscales. The DACOBS can be used to discriminate patients with psychosis from healthy respondents (Marchi et al., 2022; Müller et al., 2021; Shoham et al., 2022). It has been translated into Polish, Dutch, and Flemish versions, which have been validated in the respective associated populations (Livet et al., 2020; Whitman et al., 2002). Currently, there is not yet a DACOBS suitable for evaluating and measuring specific cognitive biases with a full‐range Likert‐type scale (ranging from 1 for strongly disagree to 7 for strongly agree) that refers to the last 2 weeks. The DACOBS aims to measure four forms of cognitive bias (jumping to conclusion; belief inflexibility bias; attention to threat bias; and external attribution bias) as well as two types of cognitive limitations (social cognition problems; and subjective cognitive problems), and avoidance behavior (safety behaviors). Our team agreed unanimously to revise the response scale to range from 0 (strongly disagree) to 7 (strongly agree) to identify a suitable cutoff point. Because the DACOBS is based on patient self‐reporting, we provide clinical‐use instructions indicating administrators should use it selectively in cases where the patient has stable symptoms, has demonstrated clear insight into their symptoms, and does not have any current long‐term or short‐term memory impairments, especially related to memory (Gawęda et al., 2018; Pena‐Garijo et al., 2022).

**TABLE 1:** Davos Assessment of Cognitive Biases Scale (DACOBS) items.

Item no.	Content
1.	I'm on the lookout for danger
2.	When things go wrong, someone is behind it
3.	I don't need long to reach a conclusion
4.	People confuse me
5.	Thoughts tend to fall apart in my mind
6.	People cannot be trusted
7.	Things went wrong in my life because of other people
8.	The right conclusion often pops into my mind
9.	I'm often not sure what people mean
10.	I pay attention to the details instead of the whole
11.	People are watching me
12.	It's NOT my fault when things go wrong in my life
13.	I don't need to consider alternatives when making a decision
14.	People surprise me with their reactions
15.	When I have a goal, I don't know how to reach it
16.	I quickly find evidence to support my beliefs
17.	People don't give me a chance to do well
18.	I make decisions faster than other people
19.	I don't understand why people react in a certain way
20.	I make sure that all windows are locked
21.	When I try to concentrate on something, it's hard to ignore other things around me
22.	I don't change my way of thinking easily
23.	I don't go to restaurants because it's not safe
24.	People make my life miserable
25.	The first thoughts are the right ones
26.	It's difficult to know what people are feeling by their facial expression
27.	I don't go out after dark
28.	I get easily distracted by irrelevant information
29.	People treat me badly for no reason
30.	I don't need to evaluate all the facts to reach a conclusion
31.	I always sit near the exit to be safe
32.	I'm not able to focus on a task
33.	People I don't know are dangerous
34.	There is usually only one explanation for a single event
35.	I don't answer phone calls, to be on the safe side
36.	I do not automatically see how things connect
37.	To protect myself, I remain on guard
38.	I don't need to look for additional information when making a decision
39.	When I hear people laughing, I think they are laughing at me
40.	It's hard to hold onto a thought
41.	I avoid considering information that will disconfirm my beliefs
42.	I don't go to shopping malls because it's not safe

The aim of this work was to produce a DACOBS that can be used to assess cognitive bias in Chinese with psychosis and to validate it in a Chinese population sample. We tested the validity and reliability of the produced modified Chinese‐language DACOBS (MCL‐DACOBS) in Chinese patients with schizophrenia. The following three hypotheses were examined: (1) The factor structure of the MCL‐DACOBS satisfies the criteria of constructive validity; (2) the MCL‐DACOBS has good reliability as demonstrated by Cronbach's *α* coefficient and intraclass correlation coefficient (ICC) values; (3) the receiver operating characteristic (ROC) curve analysis method can be used to define a clinically meaningful cutoff MCL‐DACOBS score for cognitive bias severity.

## 2 METHODS

### 2.1 Participants

This study was performed according to the criteria for the testing of medical scale validity and reliability (DeVellis, 2017; Furuno et al., 2023; Gerson et al., 1979; Kamizawa et al., 2023; Poznanovic et al., 2023; Tang et al., 2014). A total of 420 patients from the staffs of Tianjin Fourth Center Hospital and Wenzhou Seventh People's Hospital were enrolled to assess the comprehensibility of the MCL‐DACOBS. The scale's validity and reliability were assessed with 420 patients with schizophrenia from Tianjin Fourth Center Hospital, Tianjin Anding Hospital, and Wenzhou Seventh People's Hospitals between July 2021 and July 2022. All patients were admitted to the unit for at least 12 months after being diagnosed with schizophrenia according to the criteria in DSM‐IV by experienced psychiatrists who were trained in the administration of neuropsychiatric tests and used MCL‐DACOBS in their clinical practice. This work was carried out in accordance with the Declaration of Helsinki (latest version) paragraph 16, and the protocol used was approved by the local research ethics committees (Tianjin Anding Hospital, IRB No. 2020‐007 and Tianjin Fourth Center Hospital, IRB No. TW‐2022‐08‐190). All patients and volunteers provided written informed consent according to the ethics committees’ guidelines before data collection.

The patient inclusion criteria were (1) diagnosis of schizophrenia by at least two senior psychiatrists, (2) age ≥18 years, (3) ≥12 years education, (4) ability to understand the MCL‐DACOBS, (5) full insight about the disease, and (6) Mandarin Chinese speaking fluency. The same inclusion criteria, with the exception of the schizophrenia diagnosis, were applied in volunteer selection. The exclusion criteria for patients were (1) intellectual disability, (2) neurodegenerative disease, (3) history of a personality disorder, (4) brain trauma, and (5) any other factor that might interfere with cognition. After receiving a full description of the study, all patients and their guardians provided written consent to study participation.

#### 2.1.1 Criteria of the MCL‐DACOBS using illustrations

All participants were required to have demonstrated full insight and accurate memory at the time of the MCL‐DACOBS assessment. Insight was assessed with the VAGUS insight into psychosis scale (www.vagusonline.com), self‐report version (Gerretsen et al., 2014). Memory was assessed with the Wechsler Memory Scale (Hartman, 2009).

### 2.2 Localization and optimization

A group of 15 researchers collaborated to develop the MCL‐DACOBS; a total of 15 researchers modified and translated the English version of the DACOBS, 1 native‐English‐speaking specialist researcher back‐translated the scale, and 2 Chinese sinology researchers localized and optimized the language of the MCL‐DACOBS. A group of 42 senior psychiatrists evaluated the scale items’ comprehensibility. The aforementioned sinologists and English‐language specialists performed further localization and optimization based on the psychiatrists’ feedback. The final version of the MCL‐DACOBS used in this study was thus derived from the harmonized English‐language version of the scale (Livet et al., 2022).

### 2.3 Constructive validity evaluation

The validity of the MCL‐DACOBS was assessed using exploratory factor analysis (EFA) and confirmatory factor analysis (CFA). Factor loading of items into structural factors was used to assess the structure of the MCL‐DACOBS. The Tucker–Lewis index (TLI), comparative fit index (CFI), and root mean square error of approximation (RMSEA) values were calculated to test the fitness of a structural model of the MCL‐DACOBS.

### 2.4 Reliability evaluation

Four hundred and twenty patients were assessed independently by 15 raters, who were blinded to the patients’ cognitive bias statuses. Internal consistency was tested using ICC, and split‐half reliability was tested using Cronbach's *α* coefficient.

### 2.5 Definition of the cutoff point

The consistency of 15 professional doctors who have worked in cognition rescue intervention for more than 20 years was taken as the clinical standard. The area under the ROC curve (AUC) method was used (Furuno et al., 2023; Poznanovic et al., 2023; Kamizawa et, al., 2023; DeVellis., 2017; Bourgin et al., 2020) to set cutoff points for cognitive bias severity, and the sensitivity and specificity of putative cutoffs were calculated.

### 2.6 Statistical analysis

Descriptive statistics, *t* tests, ICC calculation, convergent validity assessment, and normality testing were conducted using SPSS (version 23.0; IBM Corporation). To verify the structure of the MCL‐DACOBS, EFA and CFA were conducted with a weighted least squares–, mean‐, and variance‐adjusted estimator for ordinal data in Mplus (version 7.4; Schreiber, 2008; Andreassen et al., 2011; Gerretsen et al., 2014; Solikhah et al., 2023). In the CFA, acceptable model fitness was determined based on the CFI (>.90), TLI (>.90), and RMSEA (<.08), well established as effective and reliable indicators (Schreiber, 2008; Andreassen et al., 2011; Gerretsen et al., 2014; Solikhah et al., 2023), as well as the quotient minimal discrepancy divided by the degrees of freedom (<3). To investigate a hypothesized MCL‐DACOBS structure, a theoretical one‐factor (mental suffering) structure was imposed while treating age and gender as covariates. The internal consistency of the MCL‐DACOBS was assessed by calculating Cronbach's *α* coefficient. Interobserver reliability was assessed by calculating ICCs (Marchi et al., 2022; Shoham et al., 2022; Müller et al., 2021; Brown, 2006; Dunn et al., 2014; Hu et al., 1999; McGraw & Wong, 1996; Reise, 2012).

## 3 RESULTS

### 3.1 Participants

The 420 participants (229 males/191 females) were interviewed by trained psychologists (doctoral level) with experience in cognitive measurement tool administration and who worked in the Tianjin Fourth Center Hospital or the Wenzhou Seventh Peoples Hospital. All enrolled patients were asked to complete a set of assessment questionnaires. The participants had a mean age of 27.8 ± 1.2 years, a mean illness duration was 2.8 ± 1.2 years, a mean Positive and Negative Symptom Scale score of 65.43 ± 10.69, and a mean therapeutic agent (chlorpromazine equivalent) dose of 400.25 ± 100.85 mg/day.

### 3.2 Construct validity

The EFA, performed with scores from a randomly selected subsample (*n* = 420), demonstrated that the MCL‐DACOBS had a two‐factor structure, measuring social cognitive bias (16 items) and neurocognitive bias (26 items). All factor loadings were >0.4. The Kaiser–Meyer–Olkin (.900) and Bartlett's test of sphericity (*χ* ^2^ =  2034.735) values verified the appropriateness of the sample for factor analysis. The cumulative variance contribution extent was 79.86%. The following goodness‐of‐fit indices for the two‐factor structure were obtained: CFI = .948; TLI = .784; normed fit index = .896; RMSEA = .018; goodness of fit index = .891; and adjusted goodness of fit index = .771. Promax rotation demonstrated that each item had a high factor load (range, 0.432–0.774; Table 2).

**TABLE 2:** Factor loading of MCL‐Davos Assessment of Cognitive Biases Scale (DACOBS) items.

Factor	Item	Factor loading, 95% CI	*Z*	*p*
1. Jumping to conclusions bias	3	0.617, .533–.755	39.859	<.001
	8	0.742, .572–.808	38.456	<.001
	16	0.633, .435–.720	39.258	<.001
	18	0.432, .398–.511	42.52	<.001
	25	0.544, .452–.639	2.569	<.001
	30	0.574, .411–.655	44.230	<.001
2. Belief inflexibility bias	13	0.624, .557–.805	41.598	<.001
	15	0.445, .402–.569	45.444	<.001
	26	0.537, .421–.666	35.982	<.001
	34	0.589, .492–.693	46.532	<.001
	38	0.547, .488–.599	43.230	<.001
	41	0.774, .568–.880	41.258	<.001
3. Attention to threat bias	1	0.588, .475–.666	32.589	<.001
	2	0.454, .420–.577	41.258	<.001
	6	0.573, .400–.703	31.594	<.001
	10	0.555, .417–.614	42.369	<.001
	20	0.456, .397–.597	39.518	<.001
	37	0.412, .355–.580	42.853	<.001
4. External attribution bias	7	0.456, .384–.567	41.159	<.001
	12	0.558, .491–.596	39.514	<.001
	17	0.515, .419–.727	38.157	<.001
	22	0.500, .425–.659	40.357	<.001
	24	0.445, .410–.598	31.258	<.001
	29	0.448, .397–.600	39.639	<.001
5. Social cognition problems	4	0.546, .458–.765	32.589	<.001
	9	0.517, .409–.663	35.159	<.001
	11	0.595, .479–.682	42.596	<.001
	14	0.439, .428–.491	41.528	<.001
	19	0.545, .428–.668	40.367	<.001
	39	0.484, .422–.638	33.159	<.001
6. Subjective cognitive problems	5	0.642, .500–.777	42.951	<.001
	21	0.528, .459–.636	45.287	<.001
	28	0.534, .450–.628	32.594	<.001
	32	0.440, .357–.526	39.625	<.001
	36	0.625, .555–.723	38.597	<.001
	40	0.458, .409–.527	42.357	<.001
7. Safety behaviors	23	0.557, .420–.722	45.897	<.001
	27	0.511, .485–.597	42.625	<.001
	31	0.634, .525–.699	36.258	<.001
	33	0.650, .499–.668	37.585	<.001
	35	0.591, .500–.644	33.692	<.001
	42	0.628, .600–.757	40.597	<.001

Abbreviation: CI, confidence interval.

### 3.3 Reliability

The scale had good adaptability, as demonstrated by the total ICC value of the interrater consistency (.957). Good split‐half reliability was demonstrated by Cronbach's *α* coefficient of the total scale (.965) (Dunn et al., 2014; McGraw & Wong, 1996; Reise, 2012).

### 3.4 Cutoff points

Using the clinical evaluation standard of the severity of the cognitive bias features as a reference, ROC curve analysis demonstrated that cutoff points of ≥20 and ≥10 identified cases of severe and mild cognitive bias, respectively, and with very good sensitivity and good specificity. The AUC, 95% confidence interval, sensitivity, and specificity values are reported in Table 3. Accordingly, MCL‐DACOBS scores of 11–19 were defined as indicating moderate cognitive bias.

**TABLE 3:** MCL‐Davos Assessment of Cognitive Biases Scale (DACOBS) cutoff scores.

Cutoff score	Sensitivity	Specificity	AUC, 95% CI
20	0.957	0.843	.884, .700–.900
10	0.945	0.820	.888, .747–.967

Abbreviations: AUC, area under the receiver operating characteristic curve; CI, confidence interval.

## 4 DISCUSSION

The MCL‐DACOBS was demonstrated to have ideal psychometric properties in Chinese patients with schizophrenia, with good constructive validity and reliability for identifying cognitive bias severity (Horton et al., 1987). We defined MCL‐DACOBS score ranges for three grades of cognitive bias severity. Our data demonstrated that our modified scoring did not affect the constructive validity of the MCL‐DACOBS; rather, its reliability was better than the reliabilities that have been determined for other language versions of the DACOBS. The present findings indicate that it is feasible to use the presently developed MCL‐DACOBS to assess the cognitive bias of Chinese patients with schizophrenia. To avoid the impact of biases introduced by patient‐reported content on our study, we required that the patients undergoing MCL‐DACOBS assessment should have full insight and good memory. Hence, by this method, the MCL‐DACOBS was designed to assure assessment accuracy.

Regarding the discriminant power of the MCL‐DACOBS, our data indicate that schizophrenia patients with mild, moderate, and severe cognitive biases can be identified based on total MCL‐DACOBS scores of ≤10, 11–19, and ≥20, respectively, in agreement with the results of van der Gaag et al. (2013). These cutoff points were affirmed by the clinical experience of senior physicians who have worked in cognitive rescue for more than 20 years.

The time and effort that we invested into translating the English‐language version DACOBS into the MCL‐DACOBS in the present work was fully consistent with the challenges of developing cross‐cultural assessment tools, recognizing that cognitive bias is influenced more severely by culture than by other indices. Consequently, results should be explained carefully when using the current version of the MCL‐DACOBS. Assessing cognitive bias in patients with schizophrenia may facilitate therapy to improve their cognitive performance, thus improving clinical outcomes. Further studies of the MCL‐DACOBS should be conducted to improve its clinical utility in the Chinese medical context.

### 4.1 Limitations

This study has two notable limitations. First, we used a self‐report questionnaire. Such self‐reporting, by its nature, can be affected by hiding, social desirability, and misunderstanding. Task‐based tests are better for obtaining empirical evidence of a specific cognitive bias. Second, the protocol did not include test–retest reliability. Reproducibility over time (test–retest) is one of several ways to classify and measure reliability, as is internal consistency (Banks & Marotta, 2007; Carlson et al., 2021), which measures how well the individual item scores correlate with each other. The internal consistency of the MCL‐DACOBS was shown to be satisfactory in the current study.

## 5 CONCLUSION

The MCL‐DACOBS has good validity, good reliability, and its psychometric properties indicate that it is a valid tool for measuring cognitive biases in Chinese patients with schizophrenia.

## CONFLICT OF INTEREST STATEMENT

The authors declare that they have no conflicts of interest.

## Data Availability

The original contributions presented in this study are included in the article. Further inquiries can be directed to the corresponding authors.
